# Detection and Fate of Microplastics and Nanoplastics and Technologies for Their Removal

**DOI:** 10.3390/molecules31040613

**Published:** 2026-02-10

**Authors:** Qiuping Zhang, Qi Wang, Jifei Xu, Jianguo Liu

**Affiliations:** 1 School of Life Science, Shanxi Normal University, Taiyuan 030031, China; wangqi_031230@sina.com; 2 School of Ecology and Environment, Inner Mongolia University, Hohhot 010021, China; jifeixu@imu.edu.cn; 3 School of Resources and Environmental Engineering, Inner Mongolia University of Technology, Hohhot 010051, China; liujianguo81@imut.edu.cn

**Keywords:** microplastics, nanoplastics, environment, detection

## Abstract

As primary degradation products of persistent plastic waste, microplastics (MPs, <5 mm) and nanoplastics (NPs, <1 μm) have emerged as a critical global environmental concern, with their ubiquitous distribution documented across aquatic, terrestrial, and atmospheric ecosystems. With annual plastic production exceeding 460 million metric tons, their widespread presence in environmental matrices and biota—from marine organisms to human tissues—poses significant, yet incompletely understood, threats to ecological integrity and public health. This paper systematically reviews the state-of-the-art detection techniques, environmental fate processes, and remediation strategies for MPs and NPs. In terms of detection, we cover microscopy, mass spectrometry, flow cytometry, chromatography, and spectroscopy, emphasizing hyphenated techniques (e.g., FT-IR microscopy, Raman spectroscopy) for enhancing sensitivity and specificity. Fate studies reveal that MPs/NPs exhibit long environmental persistence, undergo bioaccumulation and trophic transfer, and can act as carriers for organic pollutants and heavy metals. Removal techniques include physical (membrane filtration, adsorption), chemical (coagulation, advanced oxidation), and biological (biochar immobilization, microbial degradation) approaches, each with distinct advantages and limitations. This review synthesizes current knowledge gaps and provides a scientific framework for developing integrated management strategies to mitigate plastic pollution risks.

## 1. Introduction

Human daily life is highly dependent on plastics, leading to an exponential increase in plastic production. Due to the inability to easily obtain cheap alternatives, it is currently impossible to completely abandon the use of plastics. Therefore, plastic particle pollution will increase and expand to various parts of our ecosystem. In 2023, global plastic production exceeded 400 million tons (data from the United Nations Environment Programme), doubling compared to 20 years ago. Each year, over 300 million tons of plastic waste is generated globally, including 30 billion plastic bottles and 5 billion plastic bags [1]. Only about 14% of the plastic is recycled, with the rest entering the natural environment or landfills. Although most plastics are non-biodegradable, through physical wear caused by ultraviolet radiation and chemical processes such as photodegradation, their volume can gradually decrease. Microplastics (MPs) are usually formed by the degradation of larger plastic items, plastic particles with a diameter of less than 5 mm, or tiny particles directly produced during the manufacturing process. Nanoplastics (NPs) refer to plastic particles with a diameter of less than 1 micrometer, which are usually the products of further degradation of microplastics, extremely small in size, and difficult to recognize with the naked eye. The existence of microplastics and nanoplastics has become a global environmental issue of concern. With the increase in plastic usage, the problems of microplastic and nanoplastic pollution have become increasingly serious, and measures for monitoring and governance must be taken urgently to protect the ecological environment and human health [1].

In terms of detection, current analytical techniques primarily include microscopy, mass spectrometry, flow cytometry, chromatography, and spectroscopy, with hyphenated techniques (e.g., FT-IR microscopy, Raman spectroscopy) increasingly adopted to enhance detection accuracy. Among them, combined techniques have significant advantages in improving detection accuracy and efficiency, providing key technical support for identifying and quantifying MPs/NPs in environmental and biological samples. These detection methods not only help clarify the current pollution situation but also lay the foundation for further exploration of their environmental behavior and ecological risks. After clarifying their storage state, studies on environmental destinations show that MPs and NPs have high mobility and persistence, easily accumulating in water bodies, soil, and sediments, and can be ingested by organisms, and thus transmitted through the food chain, posing potential risks to ecosystems and human health. Understanding the migration and transformation processes and biological accumulation of MPs and NPs is a prerequisite for assessing their actual hazards and designing targeted governance strategies. Based on the understanding of their environmental behavior, multiple paths for removal technologies have been developed, such as physical methods (such as membrane filtration, adsorption), chemical methods (such as coagulation, photocatalysis), and biological methods (such as biochar, microbial degradation). Each method has its own characteristics, but no unified efficient, economical, and environmentally friendly solution has been formed, highlighting the multiple challenges in this field from theory to application.

The significance of this review lies in systematically integrating the latest research progress in the detection, environmental behavior, and governance of MPs and NPs, and investigating the key technologies, knowledge gaps, and research needs for their detection, location, and removal. It not only reveals the key technical bottlenecks and development directions in this field but also provides important references for subsequent scientific research, technology development, and policy formulation, emphasizing the necessity of interdisciplinary integration and whole-process management. By promoting the establishment of a complete technical system from precise detection to efficient removal, this review is expected to provide systematic scientific support for the effective prevention and control of plastic pollution and ecological security.

## 2. Detection

The detection and characterization of microplastics generally involve three steps: (1) extraction from the matrix [2]; (2) separation, quantification, and size determination; and (3) identification of microplastics [3]. Sensitive methods are required for the identification and quantification of microplastics. The methods employed can be classified into microscopic detection, mass spectrometry, flow cytometry, chromatographic identification, spectral analysis, etc. Each method has its own advantages and disadvantages, and the current mainstream strategy for the analysis of microplastics and nanoplastics is the combined use of multiple techniques. Generally, microscopy or flow cytometry is used for rapid screening to obtain the particle number and size distribution. For suspicious particles identified through screening, μ-FT-IR or μ-Raman is used for chemical identification. When the goal is to quantify the total mass concentration of plastics in a sample or to characterize nanoplastics (which are challenging to detect via optical methods due to their submicron size), pyrolysis–gas chromatography–mass spectrometry (Py-GC/MS) is essential. This technique enables both the identification of plastic polymers through thermal decomposition and the precise quantification of their mass, addressing the limitations of microscopy-based methods that struggle with nanoplastics and bulk mass analysis. Moreover, international guidelines have been established to ensure methodological rigor. These include protocols for air sampling of microplastics, extraction of nanoplastics from environmental matrices, and quality control in microplastic analysis. These standards provide a unified framework for sample collection, processing, and validation, enhancing the reproducibility of our results.

### 2.1. Microscopic Detection

Microscopic detection is a commonly used method for detecting microplastics, and its intuitiveness is unparalleled by any other technology [4]. Its primary value lies in revealing morphological and structural information about the microscopic world and making selections based on the requirements for the resolution, viability, and chemical composition of different research objectives. Microscopic detection generally refers to observations made using optical microscopes or electron microscopes. This method can directly obtain physical information such as the size, shape (e.g., fibers, fragments, particles), color, and surface roughness of microplastics, which is an advantage that is difficult to replicate with other technologies. Additionally, the equipment cost of optical microscopes is significantly lower than that of high-end spectrometers or mass spectrometers [5]. However, manual counting and measurement are inefficient, and the results are susceptible to subjective judgment. The resolution of optical microscopes is limited (approximately 1–2 µm), making it difficult to reliably identify sub-micron and nano-scale plastics [6]. Although scanning electron microscope (SEM) have higher resolution, they have strict requirements for sample preparation and are not suitable for rapid screening of large-volume samples. When an SEM is equipped with an energy-dispersive spectrometer, it can analyze the elemental composition of the particle surface while observing the morphology, which helps to distinguish microplastics (mainly containing C, H, and O) from natural organic or inorganic substances. Nevertheless, it is still impossible to confirm whether a particle is plastic based solely on its morphology. For example, cotton, diatoms, etc., are easily misjudged as microplastics. Moreover, the microscopic method can only provide information on particle quantity and size and cannot measure the mass concentration of polymers.

For environmental samples (such as water bodies, sediments, and digested biological tissue fluids), optical microscopes can be employed to rapidly conduct qualitative and semi-quantitative analyses of relatively large-sized (usually > 50 µm) microplastics, screen out suspicious particles, and narrow the scope of subsequent analyses. By observing the size, shape (e.g., fibers, fragments, films, particles), color, and opacity of plastic particles, the sources of microplastics can be traced. The resolution limit of traditional optical microscopes is approximately 200 nanometers (0.2 µm), which means that NPs cannot be directly observed and identified at all. Even for MPs smaller than 10 µm, observing and accurately counting them are extremely difficult [7]. A stereomicroscope can be used for operations such as classification by color, shape, and size and for preliminary screening to pick out particles. Optical microscopes are also widely used for identifying fluorescent or colored magnetic nanoparticles. SalesRibeiro et al. [4] used a confocal microscope to study the effects of microplastics on fish organs. Molenaar et al. [5] used Nile red dye to fluorescently label nanoparticles, and this method can detect nanoparticle concentrations as low as 2 × 10^6^ particles/mL.

Electron microscopes (mainly including scanning electron microscopes (SEM) and transmission electron microscopes (TEM)) are mainly used in microplastic research for situations requiring high resolution, high magnification, and surface elemental information [8]. They are not usually the first-choice screening tools, but rather, technologies for in-depth characterization of specific targets. Electron microscopes can accurately identify nanoplastics, comprehensively characterize the surface morphology and aging process of microplastics, and analyze the size distribution and shape of particles. Electron microscopes (EM) can provide information on the core size, morphology, and behavioral characteristics of particles in solution. Therefore, DLS and EM are often used in combination to verify the physical and chemical properties of MNPs. By combining scanning electron microscopy (SEM) with energy-dispersive X-ray spectroscopy and elemental distribution mapping techniques, magnetic nanoparticles (MNPs) in sediments can be detected [6]. However, the SEM/EDS technology still has problems such as high equipment cost and difficult sample preparation [7].

### 2.2. Mass Spectrometry Analysis

Mass spectrometry (MS), with its outstanding sensitivity, precision, and versatility, has emerged as the preferred analytical method for detecting MPs and NPs [9]. Different from traditional spectroscopic techniques, mass spectrometry can simultaneously determine particle size, mass, and elemental composition, enabling accurate detection even at ultra-trace concentrations in the environment [8,9,10]. For example, single-particle inductively coupled plasma mass spectrometry (sp-ICP-MS) can determine the size distribution and elemental composition of individual nanoplastics, while matrix-assisted laser desorption/ionization–time-of-flight mass spectrometry (MALDI-TOF MS) provides high-resolution mass measurements for polymer identification [11,12]. The combination of mass spectrometry and thermal analysis has been proven to be an effective method for detecting microplastics in food samples [12]. This method pyrolyzes or digests the entire sample, and the detection results are not affected by the size of plastic particles, making it particularly suitable for studying the total amount of plastic pollution in complex environmental samples. In addition to analyzing the polymers themselves, mass spectrometry can also analyze additives in plastics (such as plasticizers and antioxidants), providing more information for source tracing. However, the sample is completely destroyed during the pyrolysis process and cannot be recovered. Meanwhile, this method loses physical information such as the size, shape, and color of individual particles, and the equipment is expensive and the operation is complex. Moreover, a large amount of natural organic matter in environmental samples may interfere with the identification of characteristic pyrolysis products of polymers, requiring complex pre-treatment steps for depuration.

Advanced analytical methods based on inductively coupled plasma mass spectrometry (ICP-MS), such as single-particle inductively coupled plasma mass spectrometry (SP-ICP-MS) and inductively coupled plasma time-of-flight mass spectrometry (ICP-TOF-MS), are particularly well-suited for real-time single-particle analysis of MPs and NPs in complex environmental matrices (e.g., water, soil, biota). SP-ICP-MS enables rapid detection of individual NPs (down to ~10 nm) with high throughput (up to 10,000 particles per minute) and reproducible size/elemental composition measurements (relative standard deviation < 5% for particle count) [10], while ICP-TOF-MS offers simultaneous multi-element analysis of MPs/NPs, allowing for differentiation between plastic particles and natural colloids in mixed samples [13]. In addition, the combination of laser ablation–inductively coupled plasma mass spectrometry (LA-ICP-MS) and metal nanoparticle labeling technology can achieve accurate surface characterization and detection of nanoplastics with an extremely low error, surpassing traditional Fourier transform infrared spectroscopy (FT-IR) or Raman microscopy in terms of sensitivity and applicability [10,14]. Therefore, mass spectrometry technology has become the gold standard for comprehensive research on microplastics, effectively overcoming the limitations of other analytical methods [15].

### 2.3. Flow Cytometry

**Figure 1:**
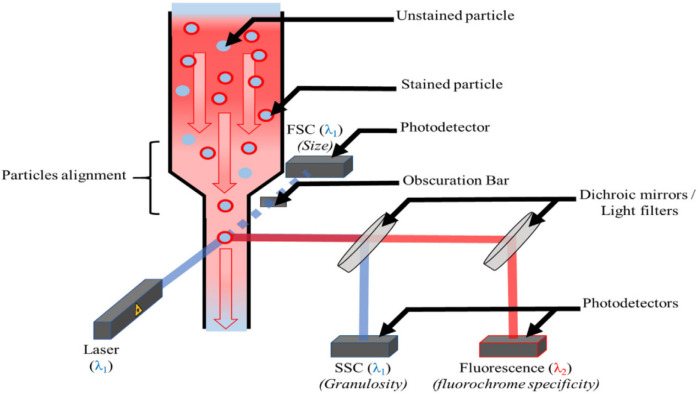
Schematic diagram of a flow cytometer, including particle alignment, measurement of dispersion parameters (FSC and SSC), and measurement of fluorescence parameters. Reproduced with permission from ref. [24].

Flow cytometry (FC) is an analytical method that utilizes the interaction between radiation and matter to detect, identify, quantify, or classify particles in a liquid medium [16]. A schematic diagram of FC is shown in Figure 1. Flow cytometry possesses high-speed and high-throughput capabilities, enabling the analysis of thousands of particles within minutes and efficient statistical analysis of particle size distribution. If equipped with a sorting function, it can sort specific particles based on light scattering or fluorescence signals for subsequent offline analysis (such as Raman spectroscopy confirmation). Currently, fluorescence staining technology (FC) is mainly applied in the field of microparticle analysis and does not require the use of nanoscale fluorescent dyes (NR) [17,18,19,20,21]. Flow cytometry (FC) can effectively detect particles with a size ranging from 1 μm to 100 μm. Due to the significant differences in the size and morphology of plastic particles, they are often difficult to distinguish from other potential particles during the initial screening. To address this issue, researchers have optimized the detection method by introducing nanoscale fluorescent dye staining technology [22,23,24]. The innovation of this technology lies in the fact that only particles with nanoscale fluorescent molecules on their surfaces emit fluorescent signals, thus enabling effective differentiation from other particles. MNP labeling technology can also enhance the fluorescent signal, enabling effective differentiation between background noise and other unstained particles (especially nanoparticles), as the diffusion signals of nanoparticles are weak due to their small size.

### 2.4. Chromatographic Determination

Chromatography (such as high-performance liquid chromatography, HPLC) has high identification sensitivity and strong separation ability, and is suitable for the quantitative analysis of polymers soluble in specific solvents (such as polystyrene (PS), soluble in tetrahydrofuran (THF)) [25,26]. However, this method dissolves or alters the sample during the analysis process and cannot provide any physical information about the particles (such as size, shape, and quantity) at all [27]. Its pre-treatment steps are complex, requiring complete extraction and dissolution of plastics from the environmental matrix. A high recovery rate is difficult to guarantee, and it is difficult to find suitable solvents for many common plastics (such as polyethylene (PE) and polypropylene (PP)) at room temperature, which limits the application scope of this method [28]. Chromatography technology is a powerful tool for detecting microplastics and nanoplastics, especially for qualitative and quantitative analysis and polymer type identification. It makes up for the deficiency of microscopy technology, which can provide physical morphology information but not chemical information [29]. The basic principle of chromatography is to allow the mixture to be tested (such as degraded plastics) to pass through the stationary phase under the drive of the mobile phase (gas or liquid). Since different components have different partition coefficients between the two phases, separation is achieved. In plastic micro-analysis, it is usually necessary to first convert solid plastic polymers into volatile small-molecule products through pyrolysis technology and then infer the type and quantity of the original polymers based on these characteristic small molecules [30]. Pyrolysis–gas chromatography–mass spectrometry (Py-GC/MS) is highly favored for its high efficiency in polymer chemical composition analysis [31,32].

**Figure 2:**
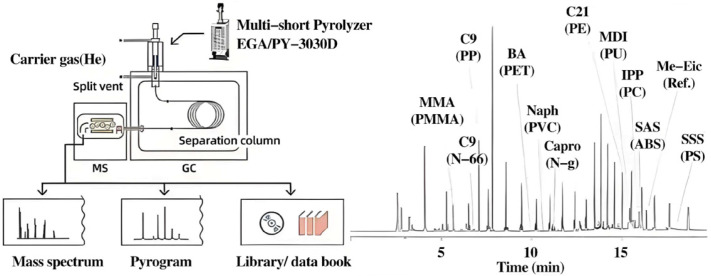
Analysis of micro/nanoparticles using pyrolysis–gas chromatography–mass spectrometry (pyrolysis-GC-MS) technology. Reproduced with permission from ref. [29].

Wu et al. used the thermal desorption gas chromatography/mass spectrometry pyrolysis method to quantitatively track microplastics in sewage sludge [26]. The research results of the Primpke team fully demonstrated the superiority of this technology [27]. The researchers compared pyrolysis–gas chromatography–mass spectrometry with hyperspectral FT-IR imaging spectroscopy for particle identification. The results showed that the Py-GC/MS technique had significant advantages in identifying specific particles. Py-GC/MS has been widely used in the analysis of complex environmental samples (such as processed seafood) after in-depth cleaning treatment to optimize the detection effect by removing organic matter and reducing the microplastic concentration (Figure 2) [28,29]. Mintenig et al. adopted the cross-flow ultrafiltration, AF4, and Py-GC/MS techniques [30]. They developed a method for analyzing nanoparticles in water environment samples. Wahl et al. [31] showed that AF4 could be combined with Py-GC/MS to detect nanoparticles in environmental samples exposed to natural organic matter (NOM), especially in soils containing plastic debris. Huiru Li et al. [32] proposed a method of dilute-HCl-assisted extraction and gel permeation chromatography–ultraviolet detection (GPC-UV) for analyzing PS-MPs in soil. The existence of MPs in the soil was confirmed through a study using scanning electron microscopy combined with energy-dispersive spectroscopy. PS-MPs were separated from the soil by stirring with a diluted HCl solution, filtering the resulting liquid, and dissolving the residue on the filter with THF.

### 2.5. Spectral Analysis

Spectral analysis is the core technique for chemical identification in current microplastic research, mainly including Fourier transform infrared spectroscopy (FT-IR) and Raman spectroscopy [33]. By comparing the results with standard spectral libraries, the types of polymers (such as polyethylene (PE), polypropylene (PP), polystyrene (PS), etc.) can be accurately identified. This analysis process does not damage the samples, and the same particle can be used for subsequent analysis. Micro-Fourier transform infrared spectroscopy (μ-FT-IR) and micro-Raman spectroscopy (μ-Raman) combine spectral and microscopic imaging functions, enabling “what you see is what you analyze” [34]. They can be employed to perform positioning analysis on selected particles and measure their sizes. However, the acquisition of spectra for a single particle is time-consuming, and a comprehensive analysis of samples containing a large number of particles is extremely time-consuming. Although automation technologies (such as focal plane array detectors) can improve efficiency, the equipment cost is relatively high. Raman spectroscopy has a high spatial resolution (about 1 µm) and can detect smaller particles, but it is extremely sensitive to fluorescence interference. Pigments or impurities in environmental samples can easily lead to a strong fluorescent background, masking the Raman signal. FT-IR is limited by the diffraction limit and has difficulty in identifying particles smaller than 10–20 µm, especially for particles smaller than the wavelength, whose signals are relatively weak.

FT-IR technology includes two different sample preparation methods: attenuated total reflection (ATR) and transmission reflection [33]. The advantage of micro-Fourier transform infrared spectroscopy (micro-FT-IR) technology lies not only in its ability to identify microplastics (MPs), but also in its role as an effective means for detecting polymer additives, providing new ideas for addressing environmental issues that threaten the health of marine life and humans [34]. Jenner et al. [35] used μ-FT-IR spectroscopy to detect microplastics in human lung tissue, analyzing digested human lung tissue samples (n = 13) to detect and characterize any existing MPs. Xu et al. [36] used surface-enhanced Raman spectroscopy (SERS) on Klarite substrates to detect and identify (single) microplastics and nanoplastics, demonstrating that surface-enhanced Raman spectroscopy is helpful for detecting microplastics smaller than 1 μm in the environment.

## 3. Fate

The fate of microplastics and nanoplastics is a dynamic global cycle, and can be divided into their migration and transformation in the environment and their fate within organisms. They continuously migrate and exchange among different environmental media (water, soil, air, and organisms), and there is almost no “final” vanishing point [37,38,39]. Their ultimate destinations are mainly the sediments of various environmental media (especially marine and freshwater sediments) and terrestrial soils. These places have become long-term “storage repositories” for plastic pollution. Due to their persistence, the sequestered plastics may re-enter the ecological cycle after hundreds or even thousands of years due to natural disturbances or human activities, posing a persistent environmental problem. Microplastics may also undergo complex transformation processes (such as aging, fragmentation, and aggregation) and interact with environmental matrices or organisms such as bacteria and pollutants [40].

The transformation processes of various nanoparticles in the aquatic environment are relatively well-defined [41]. Regarding microplastics, currently, only information about the weathering of polymers through ultraviolet photo-oxidation is available [42]. This process increases their surface area and surface exposure, which has been experimentally observed to reduce the release rate of adsorbed pollutants (e.g., heavy metals, organic contaminants) by enhancing the formation of oxygen-containing functional groups (e.g., hydroxyl, carbonyl) that strengthen pollutant–particle binding [43,44]. However, there is still a lack of understanding of the types, rates, and expected degrees of transformation of nanoplastics and microplastics in the environment [41,42,43,44]. Microplastics, with their high surface area, curvature characteristics, chemical activity, and tiny size, exhibit differentiated adsorption rates and biological distribution characteristics [42]. This dynamic property enables them to remain continuously active in the environment, constantly changing their bioavailability. The high-accumulation property of plastic materials not only makes them a transport medium for pollutants but may also make them a potential source of pollutants. When microplastics degrade into smaller particles, their surface area increases significantly, allowing them to adsorb more pollutants, including persistent organic pollutants (POPs), bioaccumulative substances, and toxic substances [45].

Ingestion is the primary way for organisms to come into contact with microplastics. Plastic ingestion has been found in humans, mammals, cetaceans, and birds. In addition to the physical harm caused by the ingestion of microplastics by organisms, these microplastics themselves may carry biomolecules that interact with biological systems or serve as a pathway for persistent organic pollutants (POPs) to penetrate into organism tissues [41]. The team of Rodriguez-Seijo [46] found signs of damage in the intestines of earthworms, manifested as damaged epithelial tissues and intestinal-wall inflammation, which may be a signal of the accumulation of polyethylene microplastics in the body. Due to their relatively large particle volume, these microplastics cause harm to organisms. The study also found that when the earthworm Eisenia fetida was exposed to polyurethane foam for 28 days, bioaccumulation of polybrominated diphenyl ethers (PBDEs) was detected in its body [47]. Lusher et al. [48] were the first to report the presence of microplastics in adult female True’s beaked whales (Mesoplodon mirus). Phthalates are used as plasticizers to soften plastic products. Studies have shown their presence in human breast milk, blood, and urine [49,50,51]. Although this is not direct evidence of the presence of plastic particles in biological fluids, it provides a direction for subsequent research. Possible routes of microplastic ingestion include inhaling microfibers in the air or ingesting them through microfibers deposited in food. These fibers can also cause lesions in the respiratory system. An increase in cancer incidence has been observed among synthetic-textile workers, and respiratory problems have occurred among workers exposed to polyvinyl chloride (PVC). Although these workers may also be exposed to high amounts of organic solvents, the potential chronic exposure to airborne microplastics may lead to lung damage, depending on individual susceptibility and particle characteristics. However, further research is needed to evaluate this [52]. To comprehensively understand the impact of microplastics on organisms, it is necessary to conduct in-depth research on their purification mechanisms. By setting up exposure experiments with different purification cycles in the laboratory, it can be verified whether animals can completely excrete microplastics or if these microplastics will remain in the body and eventually accumulate in different organs or tissues. This research is crucial as it helps us determine whether the long-term purification of shellfish will increase the risk of other animals in the food chain or humans ingesting these microplastics [44].

Purification usually refers to the process of clearing intestinal contents through defecation when there is no food intake [53]. This mechanism is crucial for understanding the accumulation of nanoplastics and microplastics as it helps to recover exposed organisms and reduce the risk of harm from these pollutants [44]. Franeke et al. reported an event similar to purification, observing that the plastic content in the stomachs of terns decreased by 80–90% within a month. They concluded that this rapid loss was mainly due to a reduction in plastic content in the birds’ gastric glands and eventual excretion.

## 4. Removal Technology

### 4.1. Physical Removal

The core challenge in physically removing microplastics and nanoplastics lies in their extremely small size, making traditional filtration and sedimentation methods insufficiently effective. The filtration technique is the most direct and widely used physical separation method, commonly employed in wastewater treatment plants and drinking water treatment plants, serving as the first line of defense against microplastics entering the environment.

Membrane filtration is an efficient physical barrier technology for removing microplastics and nanoplastics from water. Its core principle is akin to an extremely precise molecular sieve, allowing water molecules to pass through while trapping pollutants. Among common membrane processes, ultrafiltration (UF) membranes (pore size ~0.01–0.1 μm) have been reported to remove >95% of microplastics (5–5000 μm) and 80–90% of nanoplastics (1–1000 nm) in laboratory and pilot-scale studies [54], while nanofiltration (NF) and reverse osmosis (RO) membranes (pore size < 2 nm) can achieve >99.9% removal of both microplastics and nanoplastics due to their smaller pore diameters and electrostatic repulsion effects [55]. UF membranes are currently widely used in municipal and industrial water treatment for microplastic removal [54], though their application for nanoplastics is still being optimized for cost-effectiveness. Takeuchi et al. [54] demonstrated that ceramic MF membrane treatment would contribute to stable MP removal. Wan et al. [55] used gravity-driven membrane filtration, with an average water flux of 109 Lm^−2^ h^−1^, removing over 92% of model polystyrene nanoplastics beads (with an average size of 107 to 1450 nanometers). When water passes through the membrane, due to the pollutant layer blocking the membrane, it induces MPs and NPs to adsorb and accumulate on the membrane surface, reducing the efficiency of membrane filtration. By selecting selective and permeable membrane production materials, pollution can be more effectively reduced. However, several actions in wastewater treatment processes, such as choosing effective wastewater pretreatment networks and rapid membrane backwashing systems, can exacerbate the pollution problem. Therefore, to more effectively remediate plastic particles, membrane technology should be developed to address the pollution problems caused by MPs and NPs.

Sand filtration, a classic physical purification process in water treatment, captures microplastics via mechanical sieving, sedimentation, and adsorption through quartz sand’s porous structure [5]. For larger microplastics (≥10–20 μm, e.g., fibers and fragments), it achieves 50–80% removal in full-scale wastewater plants [54], acting as an important initial barrier in water purification; however, its efficiency for fine microplastics (<10 μm) and nanoplastics is typically <30% due to limited pore size and reduced gravitational effects. Therefore, although sand filtration is cost-effective and simple to operate, and plays an important role in existing facilities, it cannot be relied upon alone to solve the problem of microplastic pollution, and is usually combined with more refined deep treatment processes such as ultrafiltration membranes.

Disc filters are a physical filtration device that uses stacked filter discs for surface sieving. They form uniform pores through specific precision filter disc grooves (commonly 5–400 μm), efficiently trapping microplastics larger than this size. Simon et al. [56] demonstrated that disc filters are an efficient and reliable physical barrier, specifically when used to remove larger microplastic particles (>10 µm) in the deep treatment stage, significantly reducing the microplastic load in the final effluent. However, due to its micrometer-level filtration precision, it is largely ineffective against smaller microplastics and all nanoplastics, unable to solve the problem of microplastic pollution alone, and is mainly used as a pre-filter barrier to protect subsequent more precise treatment processes (such as ultrafiltration membranes).

The adsorption method provides an attractive technical path for removing microplastics and nanoplastics from water environments. Unlike membrane filtration, it is not prone to clogging and is not severely limited by particle size like coagulation. The removal of microplastics and nanoplastics by adsorption methods often employs a variety of porous materials. Among them, biobased adsorbents (such as chitosan, cellulose, and biochar produced from waste pyrolysis) are favored due to their low cost, widespread availability, and environmental friendliness. They effectively adsorb micro/nanoplastics through hydrophobic interactions and hydrogen bonds, but their adsorption capacity is usually limited. High-performance carbon materials (such as activated carbon) have extremely high specific surface areas and high adsorption efficiency but are more expensive. Advanced porous framework materials (such as metal–organic frameworks (MOFs)) possess well-designed pore structures and huge specific surface areas, showing extremely high adsorption potential for nanoplastics, but they are costly and their water stability issues restrict their practical application. The overall goal in research is to develop green adsorbents that have efficient adsorption capacity, are easy to separate and recycle (such as magnetic biochar), and are cost-effective. Deng et al. [57] proposed lanthanum-hydroxide carbonate nanoparticles modified with flint (LCHP) synthesized by a hydrothermal method as an effective microplastic adsorbent. Liu et al. [58] achieved efficient adsorption of homologous micro/nanoplastics in water by upgrading and reprocessing discarded surgical masks, pioneering a green water purification strategy of “treating waste with waste”. Zhang et al. [59] used discarded human hair and expanded it into micro/nanorobots for adsorption and removal of micro/nanoplastics. They first obtained a bio-hybrid micro/nanorobot based on keratin (KER) by simply decorating iron oxide microspheres with simple decoration, which has magnetic functionality for removal (MP/NPs). By integrating iron oxide microspheres, the keratin biological hybrid was regulated by an external magnetic field to achieve accurate movement and manipulation. This fuel-free keratin magnetic micro/nanorobot (KMNR) shows a significant adsorption removal efficiency of 95% and 82% in water (MP/NPs), giving it potential for multiple applications. Moreover, KMNR exhibits excellent recyclability, enhancing its sustainability. The designed KMNR has environmental and economic efficiency, and practical application attributes, providing an attractive method for addressing MP/NP pollution [59].

### 4.2. Chemical Removal

In the face of the increasingly severe challenges related to microplastic and nanoplastic pollution, chemical removal technologies have demonstrated significant advantages. The main technical approaches currently include traditional separation technologies such as coagulation–flocculation–precipitation and advanced oxidation processes based on free-radical reactions.

Coagulation, flocculation, and precipitation (CFS) is one of the most commonly used and cost-effective particle removal processes in water and wastewater treatment. Its core lies in using chemical and physical actions to aggregate dispersed tiny particles in water into larger flocs, thereby removing them through gravitational sedimentation. This process involves adding coagulants such as aluminum salts and iron salts to neutralize the negative charges usually carried by microplastics and destabilize them, and then, through flocculation, these tiny particles are aggregated into large enough flocs, ultimately achieving solid–liquid separation through gravitational sedimentation. This method has a relatively high removal efficiency for micrometer-sized microplastics and is low-cost, technologically mature, and easy to integrate into existing wastewater treatment processes. However, it is ineffective for smaller-sized nanoplastics with intense Brownian motion, and its primary goal is the phase transfer of pollutants, not achieving complete elimination. The removed microplastics eventually accumulate in sludge, posing potential secondary pollution risks. Li et al. [60] explored the feasibility and effectiveness of using the conventional water treatment process—“coagulation–flocculation–precipitation method”—to remove low-density polyethylene microplastic beads from drinking water. Peydayesh et al. [61] used coagulation–flocculation to sustainably remove MPs and natural organic matter from water through the interaction with protein amyloid fibers.

Photocatalytic oxidation represents an advanced oxidation process, utilizing the energy of light to excite semiconductor catalysts such as titanium dioxide, generating active species such as hydroxyl radicals with extremely strong oxidation capabilities. These active species can indiscriminately attack the molecular chains of plastic polymers through chain breakage and oxidation reactions, gradually degrading microplastics and nanoplastics, and the ideal product is carbon dioxide and water; this process is called mineralization. Therefore, the greatest advantage of photocatalytic technology lies in being able to completely eliminate plastic pollution at the molecular level, especially effective for nanoplastics that traditional methods find difficult to handle, and avoiding the secondary pollution problem of sludge disposal. However, its current challenges include relatively slow reaction rates, difficult catalyst recovery, and high energy consumption and costs, limiting its large-scale application. Arslan et al. [62] used n-ZnO catalysts for photocatalytic oxidation processes to remove microplastics from the effluent of urban wastewater treatment plants; Uheida et al. [63] proposed a sustainable green photocatalytic method for removing microplastics from water under visible-light activation, as a tool for removing microplastics from water. We propose a novel strategy for eliminating microplastics using glass fiber substrates to capture low-density microplastic particles parallelly supported by photocatalyst materials, such as polypropylene (PP).

### 4.3. Biological Removal

The use of biological methods to remove microplastics and nanoplastics is a very active and promising research field in environmental science. The core ideas mainly fall into two categories: one is to use biological materials as adsorbents, like magnets, to “grab” microplastics from water; the other is to achieve biodegradation, decomposing or eliminating microplastics both inside and outside the body.

Biochar has a high surface area, high porosity, a high surface-to-volume ratio and many functional groups, thus having a high affinity for pollutants and representing a promising candidate for adsorbing MPs/NPs from the environment. Abuwatfa et al. summarized the potential impacts of biochar based on its unique inherent characteristics, such as its specific surface area, porosity, functional groups, hydrophobicity and electrodynamic potential for effective removal of microplastics [64]. Wang et al. [65] found that a biochar filter has a strong ability to remove and fix microplastic spheres with a diameter of 10 µm (over 95%), and proved that biochar has great potential for immobilizing microplastic spheres (microbeads). Li et al. [66] explored the loading capacity of MPs on fresh filamentous algae and successfully synthesized magnetic filamentous algae biochar using a hydrothermal method to achieve the goal of removing MPs from water. Eamrat et al. [67] investigated the potential of chitosan extracted from shrimp shell waste (*Litopenaeus vannamei*) as a promising bioclaying agent for removing pollutants and microplastics.

The degree of biodegradability and/or microbial sensitivity of the sterilization resistance of MPs can be determined by the contamination, structural deformation, erosion, plasticizer degradation, metabolism and/or dissolution degree of MPs. The degradation of microplastics involves microorganisms such as bacteria, molds, yeasts, algae and related enzymes. Analysis and microbial technology are used to monitor the biodegradation of microplastics, but no microorganism can eliminate microplastics. Microplastic biodegradation involves fragmentation, assimilation and mineralization, influenced by abiotic and biotic factors. Environmental factors and pretreatment agents can naturally degrade large polymers or cause biological fragmentation, which may affect its efficiency. Schindler et al. [68] studied the interaction between Alternaria alternata—a fungus native to the Mediterranean—and polystyrene (PS) MPs, focusing on the potential of the fungus to remove and degrade MPs in seawater.

## 5. Conclusions

The pollution problems caused by microplastics and nanoplastics are becoming increasingly severe, and their detection and environmental behaviors, as well as technologies for their removal, have emerged as research hotspots in environmental science. In terms of detection, combining multiple analytical techniques is the mainstream strategy to improve identification accuracy and efficiency, with mass spectrometry and spectroscopy playing central roles in qualitative and quantitative analysis. Regarding environmental fate, microplastics and nanoplastics exhibit high mobility and persistence—they accumulate in sediments and organisms, transfer through food chains, and cause combined physical and chemical toxicity. For removal, physical methods like membrane filtration are efficient but prone to secondary contamination; chemical methods like photocatalysis enable complete degradation but have high costs; and biological methods like biochar adsorption and microbial degradation are green and sustainable but remain in the development stage.

To address these challenges, future research should focus on five evidence-based directions: (1) Develop standardized detection methods: Building on the current reliance on multi-technique combinations, studies should prioritize rapid, highly sensitive, and standardized protocols to enhance identification accuracy and efficiency. (2) Uncover migration mechanisms: Studies should expand on existing findings on mobility and persistence to thoroughly clarify the migration and transformation of microplastics and nanoplastics in environmental media and organisms. (3) Advance removal technology applications: Based on the trade-offs of physical, chemical, and biological methods, studies should promote practical engineering of efficient, low-cost, and eco-friendly removal technologies. (4) Strengthen source reduction: Studies should complement technical research with policy guidance and public awareness initiatives to reduce plastic pollution at its source. (5) Foster multidisciplinary collaboration: We should recognize that full-process management, supported by cross-disciplinary efforts, is fundamental to tackling the environmental challenges posed by microplastics and nanoplastics.

## Data Availability

No new data were created or analyzed in this study. Data sharing is not applicable to this article.
